# Effect of isotemporal substitution of sedentary behavior with different intensities of physical activity on the muscle function of older adults in the context of a medical center

**DOI:** 10.1186/s12877-023-03819-z

**Published:** 2023-03-07

**Authors:** Ting-Fu Lai, Yung Liao, Ming-Chun Hsueh, Kun-Pei Lin, Ding-Cheng Chan, Yung-Ming Chen, Chiung-Jung Wen

**Affiliations:** 1 Department of Health Promotion and Health Education, National Taiwan Normal University, 162, Heping East Road Section 1, Taipei 106, Taipei, Taiwan; 2 Graduate Institute of Sport, Leisure and Hospitality Management, National Taiwan Normal University, General Building 6F.,129-1, Heping East Road Section 1, Taipei City 106, Taipei, Taiwan; 3 Graduate School of Sport Sciences, Waseda University, 2-579-15 Mikajima, 359-1192 Tokorozawa City, Japan; 4 Graduate Institute of Sport Pedagogy, University of Taipei, No. 101, Sec. 2, Jhongcheng Rd., Shilin Dist, 11153 Taipei, Taiwan; 5 Department of Geriatrics and Gerontology, National Taiwan University Hospital, No.7, Chung Shan South Road, 100 Taipei, Taiwan; 6 Department of Internal Medicine, College of Medicine, National Taiwan University, No.1 Jen Ai Road Section 1, 100 Taipei, Taiwan; 7 Department of Family Medicine, College of Medicine, National Taiwan University, No.1 Jen Ai Road Section 1, 100 Taipei, Taiwan; 8 Master’s Program of Transition and Leisure Education for Individuals with Disabilities, University of Taipei, No. 101, Sec. 2, Jhongcheng Rd., Shilin Dist. 11153, Taipei, Taiwan; 9 Department of Internal Medicine, National Taiwan University Hospital, No.7, Chung Shan South Road, Taipei, 100 Taiwan

**Keywords:** Isotemporal substitution, Older adults, Muscle function, Different proportions of physical activity

## Abstract

**Background:**

Engaging in physical activity and reducing sedentary time in daily life may enable older individuals to maintain muscle mass. This study aimed to investigate the effects of replacing sedentary behavior with light physical activity (LPA) or moderate-to-vigorous physical activity (MVPA) on the muscle function of older adults at a medical center in Taiwan.

**Methods:**

We recruited 141 older adults (51.1% men; 81.1 ± 6.9 years old) and asked them to wear a triaxial accelerometer on the waist to measure their sedentary behavior and physical activity. Functional performance was assessed based on handgrip strength, Timed Up and Go (TUG) test, gait speed, and five-times-sit-to-stand test (5XSST). Isotemporal substitution analysis was performed to examine the effect of substituting 60 min of sedentary time with 60 min of LPA, MVPA, and combined LPA and MVPA in different proportions.

**Results:**

Reallocating 60 min of sedentary behavior per day to LPA was associated with better handgrip strength (Beta [B] = 1.587, 95% confidence interval [CI] = 0.706, 2.468), TUG test findings (B = -1.415, 95% CI = -2.186, -0.643), and gait speed (B = 0.042, 95% CI = 0.007, 0.078). Reallocating 60 min of sedentary behavior per day to MVPA was associated with better gait speed (B = 0.105, 95% CI = 0.018, 0.193) and 5XSST findings (B = -0.060, 95% CI = -0.117, -0.003). In addition, each 5-min increment in MVPA in the total physical activity replacing 60 min of sedentary behavior per day resulted in greater gait speed. Replacing 60 min of sedentary behavior with 30-min of LPA and 30-min of MVPA per day significantly decreased the 5XSST test time.

**Conclusion:**

Our study indicates that introducing LPA and a combination of LPA and MVPA to specifically replace sedentary behavior may help maintain muscle function in older adults.

**Supplementary Information:**

The online version contains supplementary material available at 10.1186/s12877-023-03819-z.

## Background

Decline in physical function is the primary reason for an increased risk of aging-associated disability. It is also the major contributor to the limited mobility of older adults in activities of daily living [1]. To prevent such functional disability, it is essential to reduce sedentary time and engage in physical activity every day, which may provide older persons with more opportunities to contract muscles to maintain the strength and function of their limbs [2]. However, studies have shown that the time spent in sedentary behavior (SB) increases with age [3] and is often accompanied by prolonged television viewing and telephone usage among older individuals [4]. Therefore, in free-living settings, appropriately reallocating the daily sedentary time to physical activity throughout waking time may improve the physical health of older individuals, as shown by a 15-year follow-up study [5]. This study revealed that substituting sedentary behavior with light-intensity physical activity (LPA) could have a positive effect on reducing both all-cause mortality and cardiovascular disease mortality [5].

As SB-related health risks in older adults are becoming apparent, a new World Health Organization (WHO) 2020 guideline on physical activity and SB recommends that older people limit the amount of time spent on sedentary behavior [6] and substitute it with physical activity of any intensity (LPA and moderate-to-vigorous physical activity [MVPA]). According to a systematic review, when MVPA was adjusted, the objectively measured (e.g., using ActiGraph) LPA was inversely associated with all-cause mortality risk and appeared to be associated with favorable health outcomes in the analyzed adult population [7]. Nevertheless, to the best of our knowledge, the association between LPA and muscle strength or functional capacity in older adults is not well established [8]. Although some previous reports have indicated the contribution of LPA to muscle function [2], there is limited preliminary evidence linking LPA with health benefits in older populations, such as decreased body mass index (BMI), greater handgrip strength (HGS), and self-reported lower extremity function [9–12]. More importantly, previous studies have not shown a positive correlation between the effect of replacing LPA with SB and physical functions. LPA has been proposed to act as a gateway to MVPA [13], which is strongly associated with better physical function [9, 10]. Therefore, owing to its acceptability and feasibility, LPA could be used instead of MVPA to promote more physical activity with less SB among older individuals. Consequently, it is necessary to understand whether replacing SB with LPA in conjunction with increase in MVPA enhances functional performance in older adults.

The isotemporal substitution and compositional isotemporal substitution models are novel statistical methodologies in epidemiology to investigate the potential relationship between substituting the time spent performing one activity by that for another activity [14]. In contrast, traditional multivariate regression has been used to examine the relationship of a single activity (i.e., SB) while controlling for the time spent in another activity (i.e., MVPA) as a covariate. Interestingly, a recent cross-sectional study suggested that the results stemming from these statistical approaches (isotemporal substitution and multivariate regression analysis) are generally similar, with interpretable differences in the association of variables, and warrant future research through data-oriented methodological resolution [15]. In the present study, isotemporal substitution has been defined as substituting time spent performing one intensity of physical activity with another. This enabled us to not only simulate the possible outcomes of daily behavior changes but also capture the potential health-gaining effects of lifestyle changes. Therefore, isotemporal substitution might be a more realistic approach for achieving easily interpretable and real-life public health evidence. Further, several studies on isotemporal substitution of physical functions [16–18] have shown that replacing SB with either LPA or MVPA benefitted the upper extremities (i.e., HGS). Therefore, we aimed to investigate whether replacing SB with LPA and MVPA improves the muscle strength and function of both upper and lower limbs in older adults.

## Methods

### Participants

We recruited 208 older adults aged ≥ 65 years who visited the geriatric outpatient clinic at a medical center in Taipei City, Taiwan, between September 2020 and March 2021 for treatment or health check-up. The detailed inclusion and exclusion criteria were as described in our previous study [19]. Briefly, older adults who could walk independently and had no severe hearing or visual impairment affecting communication were included. Those who lived in institutions with severe dementia or functional impairment were excluded. Of 208 participants, we excluded 15 older adults who did not undergo the full health examination and physical function checkup in the first stage. We also excluded 52 participants did not meet the criteria for wearing of the accelerometer, and 141 participants were included in the final analysis.

### Performance of physical functions

Physical function performance, including upper extremity strength (i.e., HGS), basic functional mobility (i.e., “Timed Up and Go” test, TUG) [20], gait speed (GS), and lower limb strength (i.e., five-times-sit-to-stand test, 5XSST) [21], was assessed using the BabyBot vital data recording system (Netown Corporation, Taiwan) [22]. It includes a 68-item self-reported questionnaire and tests for physical function performance [20, 21]. The HGS of the dominant hand was measured using a hydraulic hand dynamometer, with the elbow flexed while sitting. The best performance with the highest strength was selected from among three attempts, with a 1-min break between each attempt. In the TUG test, participants were instructed to rise from a standard chair, walk 3 m forward, return to the chair, and sit back down. In addition, each participant was asked to walk one-way for 6 m at their usual pace to measure the GS. A shorter time spent on the test indicated a better GS performance. Finally, lower-limb strength was measured using the 5XSST. Participants were asked to sit on a standard chair, stand up, and sit back down five times and as fast as possible.

### Physical activity and sedentary behavior

We used a waist-worn triaxial accelerometer (ActiGraph GT3X+, Pensacola, FL, USA) to measure the time spent for SB (≤ 99 counts/min), LPA (100–2019 counts/min), and MVPA (≥ 2020 counts/min) [23]. The data from this monitor were downloaded using ActiLife software (version 6.0, Pensacola, FL, USA) with 60-second epochs and a sampling frequency of 30 Hz. Using the data collection and processing criteria suggested by a systematic review of standard protocols for accelerometers [24], a period of 600 min or more of monitor wear time was defined as a valid day and zero counts of physical activity for 60 consecutive min and the sleep duration were classified as non-wear time. Sleep duration was excluded from sleep logs. Participants were requested to remove the accelerometer when they engaged in water activities, such as bathing and swimming. Data from participants who recorded at least four valid days (three weekdays and one weekend) were included in the analysis.

### Covariates

Demographic characteristics, including age, sex, education level (tertiary education or not), and living status (living alone or not), were self-reported. We calculated BMI using the weight and height formula and categorized participants as normal weight (18.5–24 kg/m^2^) or overweight (> 24 kg/m^2^). Participants were asked about their cigarette and alcohol use habits and whether they had been diagnosed with hypertension, hyperlipidemia, or diabetes (yes or no). Nutritional status was assessed using the Mini Nutritional Assessment (Short Form) [25]. The participants were categorized as having normal nutritional status (12–14 points)” or being “at risk of malnutrition (≤ 11 points).” Finally, average monitor wear time was calculated by using accelerometer and the ActiLife software.

### Statistical analyses

Descriptive analyses were conducted using the SPSS software (version 23.0; SPSS Inc., IBM, Chicago, IL, USA). Three multiple linear regression models, including the single model, partition model, and isotemporal substitution model, were used to examine the associations between SB, LPA, and MVPA for each performance of the four physical function tests. First, in the single physical activity parameter model, each activity type was assessed separately (e.g., only LPA or MVPA), without other activities, after adjusting for total wear time and covariates. Second, in the partition model, all activity types and covariates were simultaneously examined, without adjusting for the total wear time. The results of the single physical activity parameter and partition models are listed in Appendix 1. The outcomes of isotemporal substitution modeling could either be continuous or dichotomous [14]. In the final analyses, multiple linear regression models were used to examine the effect of substituting 60 min of SB with 60 min of LPA and MVPA. All activity variables (LPA and MVPA), except sedentary time, were entered into the models simultaneously, while the total wear time variable and covariates were kept constant. By including the total wear time variable, the isotemporal substitution is performed; hence, the regression estimate for each activity variable in the model reflects the effect of substituting a 60-min bout of SB with a 60-min bout of LPA and MVPA.

To simulate routine daily life, we followed the method used in previous study that used a different isotemporal substitution model [16]. The model was based on substituting 60 min of SB with 60 min of physical activity comprising both LPA and MVPA in varying proportions and increasing the duration of MVPA by 5 min every day (i.e., from 0 min/day of MVPA and 60 min/day of LPA to 60 min/day of MVPA and 0 min/day of LPA through increment of MVPA duration by 5 min/day) while controlling the total wear time and covariates.

## Results

### Participant characteristics

The analyses included 141 older adults (51.1% men; 81.1 ± 6.9 years old) (Table 1). Most participants lived with others (90.1%) and had tertiary educational attainment (61.0%). Additionally, most participants did not have a habit of cigarette use (92.9%) or alcohol use (89.4%), and the average BMI (23.7 ± 3.3 kg/m2) indicating that the participants were borderline obese. Nearly half (48.2%) of the participants had been diagnosed with hypertension, and 31.9% and 26.9% had been diagnosed with hyperlipidemia or diabetes, respectively. The average performances of the participants in HGS, TUG, GS and 5XSST activities were 23.8 ± 0.3 kg, 9.6 ± 5.7 s, 1.2 ± 0.4 m/s and 11.3 ± 5.2 s, respectively. The average durations of SB, LPA, MVPA, and wear time were 606.7 ± 74.8 min/day, 250.9 ± 79.8 min/day, 18.8 ± 26.8 min/day, and 876.6 ± 79.4 min/day, respectively.

**Table 1:** Characteristics of Participants (n = 141)

Variables	Mean ± SD	Categories	n	%
Age (years)	81.9 ± 6.9			
Sex		Men	72	51.1%
Living status		Living with others	127	90.1%
		Living alone	14	9.9%
Educational level		Lower than university	55	39.0%
		University	86	61.%
BMI (kg/m^2^)	23.7 ± 3.3	Normal	78	55.3%
		Overweight	63	44.7%
Drinking		No	126	89.4%
Smoking		No	131	92.9%
Hypertension		Yes	68	48.2%
Hyperlipidemia		Yes	45	31.9%
Diabetes		Yes	38	26.9%
Nutritional status	10.1 ± 1.2	Normal	116	82.3%
		At risk of malnutrition	25	17.7%
Handgrip strength (kg)	23.8 ± 7.5			
Timed up & go test (second)	9.6 ± 5.7			
Gait speed (m/s)	1.2 ± 0.4			
Five-time sit to stand test (s)	11.3 ± 5.2			
MVPA (min/day)	18.8 ± 26.8			
LPA (min/day)	250.9 ± 79.8			
SB (min/day)	606.7 ± 74.8			
Wear time (min/day)	876.4 ± 79.4			

SB: sedentary behavior; MVPA: moderate-to-vigorous physical activity; LPA: light physical activity

### Isotemporal substitution model

Table 2 shows the results of substituting 60 min of SB with LPA and MVPA on the physical function tests using the isotemporal substitution model. Substituting 60 min of SB per day to LPA was significantly associated with better performance in the HGS (B = 1.587, 95% CI = 0.706, 2.468), TUG (B = -1.415, 95% CI = -2.186, -0.643), and GS (B = 0.042, 95% CI = 0.007, 0.078) tests. Substituting 60 min of SB per day to MVPA was associated with better GS (B = 0.105, 95% CI = 0.018, 0.193) and lower limb strength (B = -0.060, 95% CI = -0.117, -0.003).

**Table 2:** Isotemporal Substitution Models Examining the Associations of Replacing 60 min Sedentary Behavior, LPA and MVPA on Physical Function Test (n = 141)

Analysis Method	SB			LPA			MVPA		
	B	95%CI	*p*	B	95%CI	*p*	B	95%CI	*p*
Handgrip strength (kg)									
Replace SB with	Dropped			**1.587**	**(0.706, 2.468)**	< 0.01*	1.113	(− 1.055, 3.282)	0.311
Replace LPA with	**−1.587**	**(− 2.468, − 0.706)**	< 0.01*	Dropped			−0.474	(− 2.895, 1.947)	0.699
Replace MVPA with	−1.113	(− 3.282, 1.055)	0.311	0.474	(− 1.947, 2.895)	0.699	Dropped		
Timed up & go test (s)									
Replace SB with	Dropped			**-1.415**	**(-2.186, -0.643)**	< 0.001*	-1.387	(-3.256, 0.482)	0.144
Replace LPA with	**1.415**	**(0.643, 2.186)**	< 0.001*	Dropped			0.028	(-2.070, 2.126)	0.979
Replace MVPA with	1.387	(-0.482, 3.256)	0.144	-0.028	(-2.126, 2.070)	0.979	Dropped		
Gait speed (m/s)									
Replace SB with	Dropped			**0.042**	**(0.007, 0.078)**	0.02*	**0.105**	**(0.018, 0.193)**	0.019*
Replace LPA with	**−0.042**	**(− 0.078, − 0.007)**	0.02*	Dropped			0.063	(− 0.035, 0.160)	0.204
Replace MVPA with	**−0.105**	**(− 0.193, − 0.018)**	0.019*	−0.063	(− 0.160, 0.035)	0.204	Dropped		
^a^ Five-time sit to stand test									
Replace SB with	Dropped			−0.009	(− 0.032, 0.014)	0.435	**−0.060**	**(− 0.117, − 0.003)**	0.030*
Replace LPA with	0.009	(− 0.014, 0.032)	0.435	Dropped			−0.051	(− 0.114, 0.013)	0.117
Replace MVPA with	**0.060**	**(0.003, 0.117)**	0.030*	0.051	(− 0.013, 0.114)	0.117	Dropped		

Adjusted for sociodemographics (age, sex, education, living status), health status (BMI, hyperlipidemia, hypertension, diabetes, alcohol, smoking and nutritional status.) and monitor wear time; *p < 0.05

^a^ Log-transformed

SB: sedentary behavior; MVPA: moderate-to-vigorous physical activity; LPA: light physical activity

### Mixed redistribution–substitution model

**Fig. 1:**
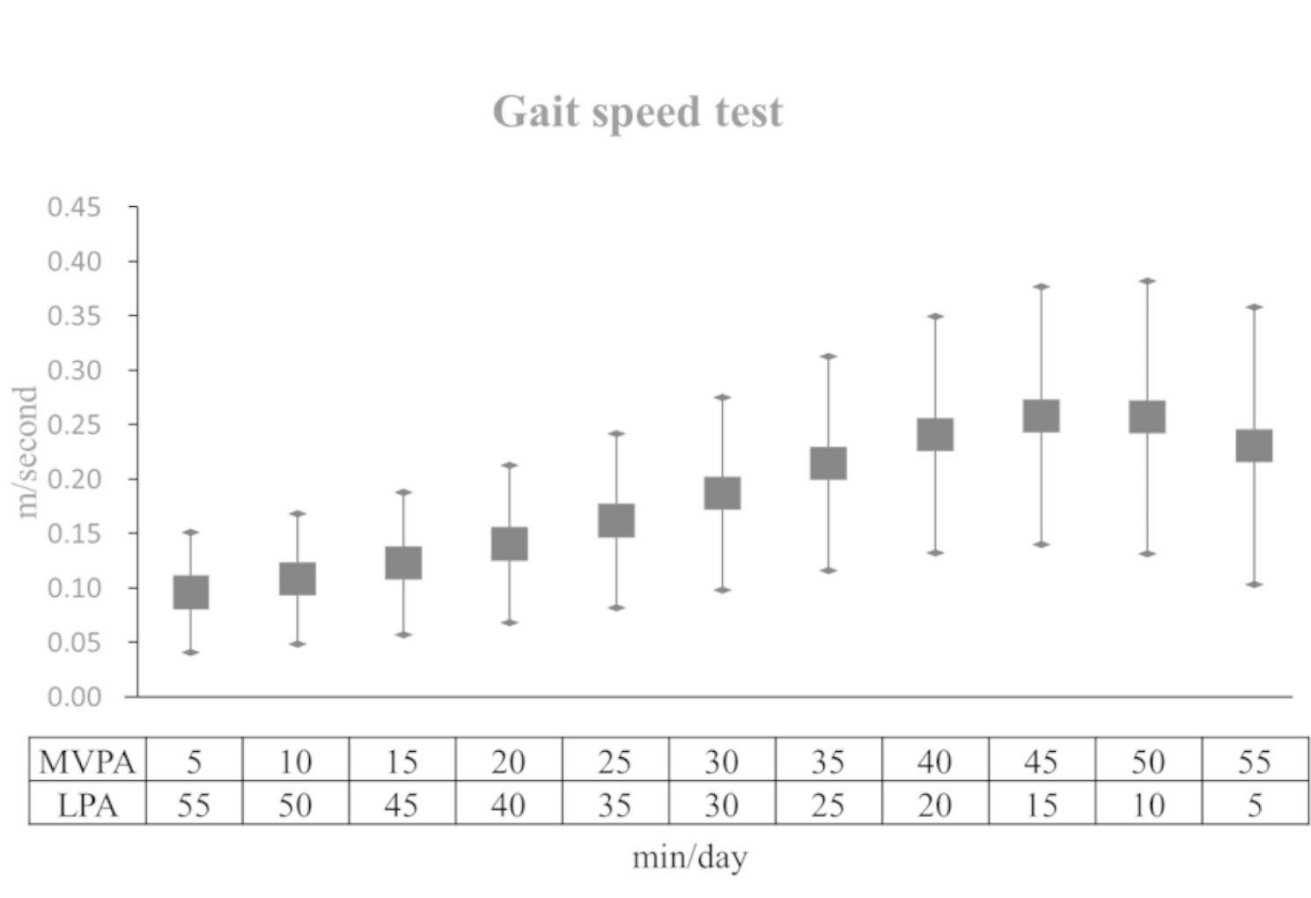
Substitution regression model for the effect of replacing SB with various ratios of LPA and MVPA on the gait speed (0–60 min). The values indicate the parameter estimate and 95% CI adjusted for patient sociodemographic characteristics (age, sex, education, living status), health status (BMI, hyperlipidemia, hypertension, diabetes, alcohol, smoking and nutritional status.), and average monitored wear time SB: sedentary behavior; MVPA: moderate-to-vigorous physical activity; LPA: light physical activity

**Fig. 2:**
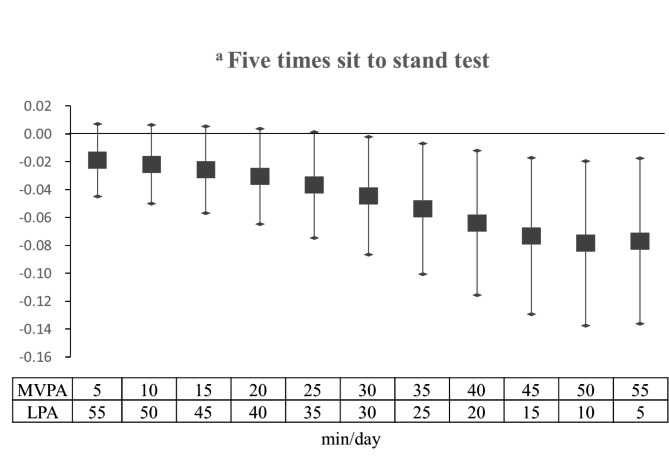
Substitution regression model for the effect of replacing SB with various ratios of LPA and MVPA on the five-times-sit-to-stand test (0–60 min). The values indicate the parameter estimate and 95% CI adjusted for patient sociodemographic characteristics (age, sex, education, living status), health status (BMI, hyperlipidemia, hypertension, diabetes, alcohol, smoking and nutritional status.), and average monitored wear time ^a^ Log-transformed SB: sedentary behavior; MVPA: moderate-to-vigorous physical activity; LPA: light physical activity

The association between substituting 60 min of SB with a combination of LPA and MVPA and the GS and 5XSST findings is presented in Figs. 1 and 2. We found that substituting SB with LPA, but not MVPA, was associated with a better performance in the HGS and TUG tests; thus, we did not perform a mixed redistribution–substitution model using either of these tests. In general, the substitution of SB with any combination of LPA and MVPA tended to improve the GS. Each 5-min increment in MVPA in the 60-min physical activity substituting SB resulted in greater GS improvement. Specifically, substituting 60 min of SB with 30 min of each LPA and MVPA per day showed statistically significant improvement and probable changes in the 5XSST (B = -0.044, 95% CI= -0.087, -0.002).

## Discussion

To our knowledge, this is the first study to demonstrate that substituting SB with 60-min LPA per day among the elderly has a positive relationship with their HGS and performance in the TUG and GS tests. This indicates that substituting 60 min of SB with LPA among older adults is mathematically associated with better muscle function in 4 limbs. Moreover, we also observed that substituting 60 min of SB with MVPA is mathematically associated with a better performance of lower limb functions (e.g., GS and 5XSST). On substituting 60 min of SB with mixed type of physical activity with LPA and MVPA, the results showed an overall positive relationship with GS.

In the past, few attempts of isotemporal substitution of SB in older adults with LPA have been reported, and they were unable to conclusively link LPA with the improvement of muscle strength and function in older adults. In our study, however, we found that replacing SB with LPA improved HGS, TUG test performance, and GS. In terms of HGS, while previous systematic reviews and meta-analyses suggest that LPA and HGS have a positive relationship, including four studies representing 3,215 individuals [2], our results indicate that a daily 60-min substitution of SB with LPA is associated with better HGS among older adults.

In addition to HGS, the function of the lower limbs was also positively correlated with the 60-min LPA substitution, as measured by the TUG and GS tests, which may imply that a lower intensity of walking has a beneficial impact on the lower limbs [2]. Previous studies on isotemporal substitution analysis to investigate the relationship between SB, physical activity, and lower limb strength and function only showed that substitution with MVPA, and not LPA, was positively correlated with improvement of lower limb function [16, 18]. In our study, we observed positive effects on the lower limb function on substituting 60-min SB with not only 60-min MVPA but also with 60-min LPA (GS test, Table 2). Specifically, our results indicate that replacing SB with LPA would suffice for improvement of lower limb function mathematically. Older age in this study (81.9 years vs. 70.7 and 74.4 years) may account for the observed differences since adults with advanced age were less like to be engaged in MVPA. [16, 18]. Previous studies have highlighted the importance of interrupting SB to prevent a decline in physical function over a 12-month follow-up [26] or in a large cohort [27]; these studies have also shown that sit-to-stand transitions may be adequate in improving lower extremity strength. Our findings may further inform the importance of replacing SB with just 1 h of LPA in the context of hospitals and relatively older adults (80 + years) and may provide greater motivation to aged individuals to undertake LPA to improve lower limb performance.

Interestingly, when SB was replaced with MVPA instead of LPA, a similar association with HGS was not observed (Table 2). We reasoned that as MVPA may be heavily reliant on lower-limb-related physical activity for its higher intensity, such as in dancing, bicycling, or running [28], the intensity of LPA may be associated with daily living activities, such as household chores, with lower intensity of walking behavior in older adults, leading to better HGS [29]. Additionally, WHO guidelines recommend an optimal MVPA duration 150 to 300 min/week for older adults [6]. Our clinically recruited participants, who were mostly aged ≥ 75 years, may have found it difficult to attain the suggested allocation. Regardless of reports suggesting that engaging in MVPA increases the risk of injury among older persons, it is our belief that increasing LPA is a reasonable and attainable strategy for improving upper limb strength in older individuals.

Our results indicate that reallocating 60 min of SB toward a combination of LPA and MVPA in different proportions tended to mathematically improve both strength and function of the lower limbs in older adults (Figs. 1 and 2). With respect to muscle function (Fig. 1), we found that replacing 60 min of SB with a combination of LPA and MVPA and increasing the proportion of MVPA by increments of 5 min was significantly associated with the GS, which is consistent with a previous report [16]. However, the lower limb strength as measured by 5XSST (Fig. 2) only showed mathematical improvement when the 60-min SB was reallocated to at least 30 min, rather than 10 min, of MVPA, as has been previously described by Lerma et al. [16]. This is likely due to the significant difference in the mean age in this study (81.9 years) and that of Lerma et al. (70.7) [16]; our cohort was older and would have needed to exert more effort to build muscles.

We and other researchers have shown that substituting SB with MVPA mathematically improves lower limb strength and function among aged persons. They may remain reluctant to perform higher intensity physical activity as suggested [30, 31], considering that in an aging society, older adults are often afflicted with comorbidities or functional decline [32]. Importantly, our findings on substituting SB with LPA, which was positively associated with upper limb strength and lower limb function, may pave the way for designing a feasible regimen of combined LPA and MVPA in varying proportions for older adults.

To the best of our knowledge, only one study has used isotemporal substitution analysis to investigate the association between substituting SB with a combination of LPA and MVPA and lower limb function [16]. Notably, this is the second such study; however, it has a larger sample size and was performed among medically enrolled older adults. Nonetheless, our study had some limitations. First, owing to the cross-sectional design of our study, we could not interpret the causality between substitutions of physical activity and function of the tested subjects. For instance, the results of the mixed redistribution–substitution model may reflect the findings of the isotemporal substitution model, which showed a positive relationship between MVPA and lower limb strength. Second, the small sample of clinically recruited participants was not representative of the community-dwelling population as a whole. Further studies with a longitudinal design and representative sample size are therefore necessary. Third, there are some drawbacks of using the hip-worn Actigraph GT3X + to measure physical activity among older people [33]. For example, in case of popular activities such as swimming, the device was not wear. Moreover, the gadget may overestimate or underestimate the SB status owing to failure in measuring body posture, which leads to the inability in detecting the physical activity of standing, lying down, and taking an afternoon nap. Finally, the cutoff point to differentiate LPA from MVPA is arbitrary. As the intensity of physical activity occurs on a continuum, it is important to carefully interpret the upper limit of what is considered LPA and the lower limit of what is considered MVPA.

## Conclusion

It is certain that reducing SB from the activities of daily living of older individuals is the key to preserving limb strength and function. We demonstrates substituting 60 min of SB with LPA boosted upper limb strength and lower limb function in older adults. In addition, replacing 60-min SB with a combination of LPA and MVPA everyday enhanced both limb strength and function of the older individuals. LPA may be more appealing to the elderly population, and our study suggests suitable alternatives to promote the physical health of older adults based on the intensity and feasibility of physical activity.

## Electronic supplementary material

Below is the link to the electronic supplementary material.

Supplementary Material 1. Appendix 1. Single Models and Partition Models Examining the Associations of SB, LPA and MVPA on Physical Function Test (n=141)

## Data Availability

The datasets used and/or analysed during the current study available from the corresponding author on reasonable request.

## List of abbreviations

SB: sedentary behavior
LPA: light physical activity
MVPA: moderate-to-vigorous physical activity
HGS: handgrip strength
GS: gait speed
TUG: Timed Up and Go
5XSST: five-times-sit-to-stand test
