# Supplementary material for: Effect of isotemporal substitution of sedentary behavior with different intensities of physical activity on the muscle function of older adults in the context of a medical center
Source: BMC Geriatr. 2023 Mar 7;23:130. doi: 10.1186/s12877-023-03819-z (PMC9993594; doi:10.1186/s12877-023-03819-z)
Supplement: Supplementary file 1 — Supplementary Material 1. Appendix 1. Single Models and Partition Models Examining the Associations of SB, LPA and MVPA on Physical Function Test (n=141) [file 12877_2023_3819_MOESM1_ESM.docx]

Appendix 1. Single Models and Partition Models Examining the Associations of SB, LPA and MVPA on Physical Function Test (n=141)

| Analysis Method | SB (h/day) | | | LPA (h/day) | | | MVPA (h/day) | | |
| --- | --- | --- | --- | --- | --- | --- | --- | --- | --- |
|  | B | 95%CI | *p* | B | 95%CI | *p* | B | 95%CI | *p* |
| Handgrip strength (kg) |  |  |  |  |  |  |  |  |  |
| Single model | **-1.564** | **(-2.367, -0.761)** | **<0.01*** | **1.676** | **(0.770, 2.582)** | **<0.01*** | 1.728 | (−0.560, 4.015) | 0.138 |
| Partition model | −0.732 | (−1.635, 0.172) | 0.111 | **0.886** | **(0.078, 1.694)** | **0.032** | 0.570 | (−0.1730, 2.870) | 0.625 |
| Timed up & go test (s) |  |  |  |  |  |  |  |  |  |
| Single model | **1.410** | **(0.729, 2.090)** | **<0.01*** | **-1.477** | **(-2.247, -0.707)** | **<0.01*** | -1.759 | (−3.704, 0.186) | 0.076 |
| Partition model | -0.004 | (−0.770, 0.762) | 0.993 | **-1.418** | **(-2.103, -0,733)** | **<0.01*** | −1.391 | (−3.340, 0.559) | 0.161 |
| Gait speed (m/s) |  |  |  |  |  |  |  |  |  |
| Single model | **-0.093** | **(-0.138, -0.049)** | **<0.01*** | **0.086** | **(0.035, 0.137)** | **<0.01*** | **0.186** | **(0.062, 0.310)** | **<0.01*** |
| Partition model | -0.012 | (−0.062, 0.037) | 0.628 | **0.067** | **(0.022, 0.111)** | **<0.01*** | **0.153** | **(0.027, 0.279)** | **0.018*** |
| ^a^ Five-time sit to stand test |  |  |  |  |  |  |  |  |  |
| Single model | **0.022** | **(0.001, 0.043)** | **<0.01*** | -0.016 | (-0.040, 0.008) | 0.181 | **-0.069** | **(−0.126, -0.011)** | **0.019*** |
| Partition model | -0.013 | (−0.037, 0.010) | 0.275 | **-0.026** | **(−0.047, -0.005)** | **0.014*** | **−0.078** | **(−0.138, -0.018)** | **0.011*** |

Adjusted for sociodemographics (age, sex, education, living status) and health status (BMI, hyperlipidemia, hypertension, diabetes, alcohol, smoking and nutritional status.); *p<0.05.

^a^ Log-transformed

SB: sedentary behavior; MVPA: moderate-to-vigorous physical activity; LPA: light physical activity
